# Influence of Cultivar on Nutritional Composition and Nutraceutical Potential of Pecan Growing in Uruguay

**DOI:** 10.3389/fnut.2022.868054

**Published:** 2022-06-22

**Authors:** Virginia Ferrari, Guillermo Gil, Horacio Heinzen, Roberto Zoppolo, Facundo Ibáñez

**Affiliations:** ^1^Laboratorio de Agroalimentos, Instituto Nacional de Investigación Agropecuaria, Canelones, Uruguay; ^2^Laboratorio de Química de Productos Naturales, Departamento de Química Orgánica, Facultad de Química, Universidad de la República, Montevideo, Uruguay; ^3^Programa Nacional de Investigación Producción Frutícola, Instituto Nacional de Investigación Agropecuaria, Canelones, Uruguay

**Keywords:** *Carya illinoinensis*, nuts, polyphenols, ORAC, tocopherols, fatty acids, antioxidant activity (AA), tannins

## Abstract

Composition and antioxidant properties of sixteen pecan [*Carya illinoinensis* (Wangenh) K. Koch] cultivars grown simultaneously in a single experimental orchard in Uruguay were evaluated to characterize their nutritional properties and nutraceutical potential. The percentage of oil, moisture, ash, minerals, and proteins were determined and also the fatty acid profile. Total phenolic compounds [18–41 mg gallic acid equivalents (GAEs)/g], condensed tannin [2–12 mg catechin equivalents (CEs)/g], and tocopherols (110–163 μg/g) contents were estimated in nut kernels. Total phenolic compounds (32–117 mg GAE/g), condensed tannins (130–357 mg CE/g), and total anthocyanins (1–3 mg 3-glucoside cyanidin/g) were also determined for pecan shells. The antioxidant activity in shells [57.15–578.88 μmol Trolox equivalents (TEs)/g] was 5 times higher compared with the kernels (23.15–156.60 μmol TEs/g) measured with hydrophilic ORAC. Bioactive compounds concentrations present statistically significant genetic variability between cultivars studied (*p* < 0.05). The presence of phenolic compounds was related with high-antioxidant capacity in kernels and shells, and a strong correlation between content of total phenolic compounds and condensed tannins in pecan shells was found. Principal component analysis (PCA) and hierarchical cluster analysis (HCA) show association between cultivars and the observed variables. The nutritional profile in the different cultivars showed the trends described in other countries, but this work shows some significant differences that could be attributed to the specific edaphoclimatic conditions of cultivation in Uruguay.

## Introduction

Pecan [*Carya illinoinensis* (Wangenh) K. Koch] or American walnut, is a specie belonging to the Juglandaceae botanical family. The fruit is a dry drupe that can appear grouped on a short peduncle, from one to four. They are oblong and ellipsoid measuring 3–5 cm long and are made up of a kernel that includes the embryos and the outer part brown testa (edible part), a nutshell (hard, smooth, thin, and brown endocarp) and an exocarp that detaches at harvest time, when the fruit ripens (1).

In Uruguay, the first commercial plantations were done during the sixties and basically to produce nuts in small scale (2). The actual planted area is estimated to be around 600 hectares The average annual relative humidity in the country is between 70 and 78% and the annual pluviometry is greater than 1,100 mm but is highly variable among seasons and years, so plantations with irrigated systems are highly recommended (3). The average annual temperature is 17.7°C, varying from 19.8°C in the northwest zone to 16.6°C in the south coast (4). Multidisciplinary investigations have been developed in the last years to prospect, characterize, and improve production of diverse genetic materials planted in Uruguay due to mainly an increasing interest in an alternative commercial culture (2, 5) but still little local information is available on the nutritional and phytochemical profile of the pecan nuts.

The phytochemical composition of nuts cultivars depends not only on the location, climatic conditions or agricultural practices but also of the cultivar genetics (6–8). To our knowledge, there is currently no study concerning the effect of genetics under the same soil, climate, and cultural conditions for a high number of cultivars. This information is important not only to characterize bioactive compounds in pecan nuts but as well for better understanding nutraceutical properties given by the high content of antioxidant compounds reported in these fruit (6, 9, 10).

In general, fixed oils in kernels are the major component, highlighting the content of mono-unsaturated fatty acids. The fatty acid profile of pecan is similar to the olive (*Olea europaea L.*) oil, which is also recognized as a healthy oil. There is a correlation of fatty acids with blood circulation and cardiovascular diseases (11), where it has been revealed that high levels of circulating linoleic acid are inversely related to the mortality associated with this type of disease. Oleic and linoleic acids have also been linked to the lower risks of type 2 diabetes, inhibiting negative regulators in the insulin pathway and linking linoleic acid to stimulating insulin secretion (12). Arachidonic, linoleic, and oleic acids markedly influence the immune response, inflammatory process, and regulate cholesterol homeostasis; their consumption has been linked to significant beneficial effects on the cardiovascular health (13).

There are also reports about phenolic compounds in foods that classify the pecan among the 20 foods with the highest content (14, 15). Within polyphenols, several types have been described in pecan, such as flavanols, anthocyanidins, proanthocyanidins, phenolic acids, and ellagic acid. Pecan has been characterized as having a high concentration of flavonoids (34 mg/100 g of seed) and also containing epigallocatechin-3-gallate (EGCG), a powerful antioxidant agent that has been shown to present favorable health effects on obesity, cardiovascular, neurological diseases, and cancer (16, 17). These compounds are readily bioavailable and added to the low content in carbohydrates (10–15%), results that pecans can be a suitable dietary supplement for people with diabetes (18). The kernel also contains proteins (8%) and food fiber (7.5%). It is a source of vitamins, mainly vitamins A, C (ascorbic acid), B1 (thiamine), B2 (riboflavin), and E, where gamma-tocopherol contents are of special interest. These compounds were identified as important nutritional factors for reproduction, representing powerful antioxidants, cell-signaling regulators, gene expression modulators, and appear to be effective in preventing cancer-related processes (16). Potassium and phosphorus minerals were found in variable concentrations among different cultivars, and pecan nuts are also considered to be a good source of zinc and manganese (18).

In many investigations, the antioxidant activity in foods was measured *in vitro* to estimate the potential antioxidant capacity and the possible benefit of including it in a human diet. But very less information is available for pecan nuts. The total antioxidant activity measured by hydrophilic ORAC of pecan seed has been reported between 120 and 245 μmol Trolox equivalents/g of seed and was significatively affected by the cultivars and postharvest management (6, 19).

The nut shell represents a percentage in weight varying between 40 and 50% of the total fruit, and some studies have shown that it also has a high-antioxidant capacity, which could be considered a new source of natural antioxidants (6, 18, 20–24) with potential in the food industry (25) and pharmacological uses (26).

The objective of this work was to study nuts of sixteen pecan cultivars to evaluate and compare their chemical and nutraceutical constituents. This information is of great interest in the present not only to characterize the nuts of each cultivar and evaluate the effect of climatic, agronomic, and cultivation factors, but also to consider the shells. This will help to determine their value as a by-product and achieve a better understanding of their potential contribution to health promotion and disease prevention.

## Materials and Methods

### Sample Preparation and Preservation

**FIGURE 1:**
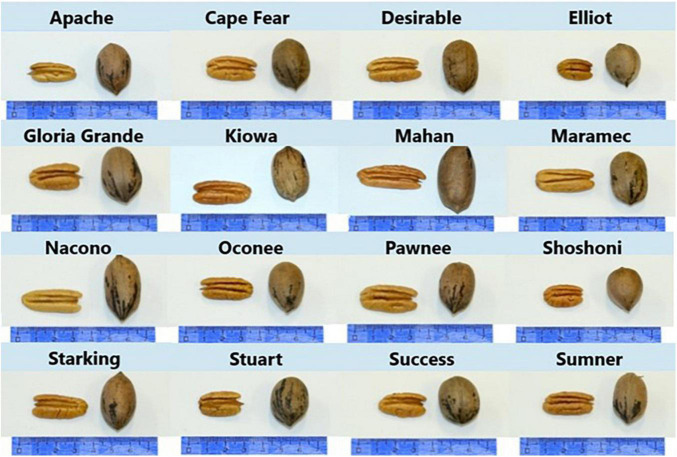
Pecan nuts of 16 cultivars growing in Uruguay, at harvest time. Scale in cm.

All the trees are being grown in an evaluation orchard at the Instituto Nacional de Investigación Agropecuaria–INIA “W. Ferreira Aldunate” Experimental Station in the South of Uruguay (S 34° 67′, W 56° 37′). According to Köppen–Geiger classification, the climate of the studied region is “Cfa” and the soil type is a Typic Argiudoll. Most of these soils have not more than 60 cm of A horizon, followed by a well-developed B clay horizon. Thus, ridges 30–40 cm high are made following the tree lines before planting. Trees were planted in 2010 and drip irrigation was installed at that time. The spontaneous vegetation in the row had one or two herbicide applications yearly, and between rows a mix of grasses and legumes was cover sown every other year. Soil cover was cut shortly before each harvest. The nuts were harvested from March through May 2019. The cultivars were Apache, Cape Fear, Desirable, Elliot, Gloria Grande, Kiowa, Maham, Maramec, Nacono, Oconee, Pawnee, Shoshoni, Starking, Stuart, Success, and Sumner (Figure 1). The cultivars are planted in field duplicates with three trees in each plot. A composite sample of each plot was taken, thus, two per cultivar, except in the case of Desirable, Mahan, and Shoshoni where only one field plot was harvested. The homogeneity of the soil and the agronomical practices allow the combination of harvest samples for each cultivar and then take over three subsamples for the chemical analysis. For every analyzes, approximately 1 kg of the harvested nuts were randomly selected to generate the laboratory subsample. They were crushed manually to separate the kernels from the shells and the weights were registered. The kernel samples were dried in an oven with an air flow at 70°C until constant weight, approximately 2 days. The kernel and shell dry samples were ground using a food processor. All the samples were stored at −80°C in the Falcon tubes.

### Proximate Composition and Minerals

To estimate moisture content in kernel and shell samples the official methodology AOAC 925.40 (27) was followed. In kernels, the extraction of the lipid fraction was carried out in accordance to the official methodology AOAC 948.22 (27). Briefly, 2 g of ground samples were extracted with hexane in a Soxhlet equipment for 6 h. After that, the remaining solvent in samples was evaporated and final weight recorded to calculate oil percentage. Kjeldahl method AOAC 981.10 (27) was used to estimate protein content based on total nitrogen determination. The total nitrogen values of each sample were multiplied by the conversion factor (6.25) to calculate total protein percentage content in each kernel sample. The official methodology AOAC 950.49 was followed to determine ash content. In total, 5–10 g of ground sample were placed in a flask at 550°C until carbonization. It was left in desiccator until room temperature was reached before final weighing and percentage of ash determined. The acid digestion of ash was carried out with 6 N hydrochloric acid. After diluting and filtering, the elements zinc, manganese, calcium, iron, magnesium, and potassium were measured by atomic absorption spectrometry (AOAC 984.27). The results were expressed in milligrams of the corresponding mineral per 100 g of kernel on wet basis.

### Fatty Acid Profile

For fatty acid profile analysis, approximately 0.1 g of ground samples and 2 ml of heptane were vortexed for 1 min, left standing for 16 h at 20°C and then centrifuged at 12,000 rpm for 5 min. The supernatant was transesterified adding 1 ml of methanol (4 N potassium hydroxide) and manually stirring for 1 min. The solution was dried with sodium sulfate, centrifugated at 12,000 rpm for 5 min. In total, 200 μl of the upper phase was diluted to 1 ml final volume, filtrated and injected into a gas chromatography equipment coupled with flame ionization detector (GC–FID, Shimadzu 2010 Plus, Japan). The column used was Agilent DB–WAX (30 m × 0.25 mm ID, 0.25 μm). The injection temperature was 250°C and the FID detector temperature was set at 300°C. The oven temperature was set at 13 min from 160 to 200°C and stay until 22 min, then the temperature increases to 240°C ending the program at 25 min. The sample injection volume was 1 μl, the mobile phase was hydrogen at 40 ml/min (split ratio at 180). Standard FAME (fatty acids methyl esters) was used for identification of the peaks and content was expressed as percentage of the total fatty acids.

### Total Phenols

Total phenols were determined according to the method adapted from Ibañez et al. (28) with Folin–Ciocalteu reagent. For this, 2 g of sample were extracted with 10 ml of 80% methanol, homogenized in Ultraturrax for 2 min and centrifuged at 10,000 rpm for 4 min at 4°C. The analysis was carried out in microplates using gallic acid as standard for quantification. Diluted sample extract (15 μl), 240 μl of distilled water, 15 μl of Folin–Ciocalteu reagent and 30 μl of carbonate 1 N sodium were incubated for 15 h in the dark. The absorbance reading was performed at 760 nm in a Synergy H1 microplate reader and analyzed using Gen 5 software (BioTek Instruments Inc., Winooski, VT, United States). The results were expressed as milligram of the gallic acid equivalents (GAEs) per g of kernel or shell.

### Condensed Tannins

Condensed tannins content was determined using the vanillin method adapted from Herald et al. (29). Briefly, 0.1 g of each sample was mixed with 2 ml methanol:hydrochloric acid (99:1). The solution was vortexed for 1 min and centrifuged at 8,000 rpm for 1 min. The color development was carried out in a 96-well microplate, 50 μl of extract each sample were incubated for 20 min with 250 μl of 1% vanillin dissolved in methanol:hydrochloric acid (92:8). For the calibration curve, a catechin solution in hydrochloric acid was used as standard. Absorbance at 500 nm was recorder using a microplate reader described previously. The results were expressed as milligrams of catechin equivalent (CE) per g of kernel or shell in wet weight basis.

### Tocopherols

For tocopherols determination the methodologies of Villareal et al. and Kruger et al. (6, 30) were adapted. Kernel pecan nut powder (2 g) was mixed with 10 ml of methanol (0.01% BHT) in Ultraturrax for 1 min. Samples were then centrifuged at 8,000 rpm for 5 min and 1 ml aliquot was filtered and 20 ml injected into HPLC–DAD (Shimadzu 20A, United States). Retention times and spectral profile were used for identification, the quantification was done at 295 nm by comparison with alpha-tocopherol calibration curve and results were expressed as mg/100 g of kernel in wet weight basis.

### Total Anthocyanins

Anthocyanins determination was made in the shell samples by the differential pH method according to a methodology adapted from Lee et al. (31). A sample of 0.2 g was extracted with 2 ml of methanol (80:20, v/v) homogenized and centrifuged at 10,000 rpm for 4 min. In 96 wells, microplates 40 μl of the extract and 200 ml of buffer solution at pH 1 (0.025 M KCl) were mixed. This last step was repeated by replacing the pH 1 buffer with the pH 4.5 buffer (CH_3_CO_2_Na_3_H_2_O; 0.4 M). After stabilization for 30 min, the absorbance was measured at maximum absorption wavelength (Abs_max_) of pH 1 extract (454 nm) and 700 nm (Abs_700_) in microplate reader described previously. Anthocyanins in extracts were estimated with the following equation:


A=(Abs-maxAbs)700,pH1-0(Abs-maxAbs)700,pH45

After that, calculation of anthocyanin concentration was based on the Beer–Lambert law and cyanidin 3-glucoside extinction coefficient (26.900 M^–1^ cm^–1^). The results were expressed as milligrams of cyanidin-3-glucoside (mg c3 g) per g of nut shells.

### Antioxidant Capacity

The study of antioxidant capacity was performed according to the methodology described in Galili and Hovav (32) by the oxygen radical absorbance capacity test (ORAC). Briefly, kernel and shell samples (0.2 g) were mixed with 1 ml of methanol (80:20, v/v). After homogenization, the samples were placed in an ultrasonic bath during 10 min at 20^°^C and stored for 4 h at 20^°^C avoiding the light exposure. After that, they were centrifuged at 12,500 rpm during 5 min. Diluted methanolic extracts or control (gallic acid) or standard solutions of Trolox (25 μl) were transferred to 96 wells-microplates. The plates were placed in the microplate reader and 150 μl of fluorescein solution (0.008 μM) prepared with 75 mM phosphate buffer were added using the automatic dispenser. After shaking and incubation at 37^°^C for 30 min, 25 μl AAPH (153 mM) prepared freshly with 75 mM phosphate buffer was added to each well. The plate was agitated, and the fluorescence was monitored continuously during 80 min every 1 min. The wavelengths were set in 485 nm for excitation and 528 nm for emission. The results were estimated based on the standard curve of Trolox concentrations and net area in the fluorescence decay curve. The ORAC activity was expressed as μmol Trolox equivalents (TE) per g of sample.

### Statistical Analysis

All the data are reported as mean of three replicates, except percentage of kernel in nut weights. To determine statistical differences between cultivars ANOVA analysis of variance was applied (*p* < 0,05) in Infostat Software (33). Statistical error of mean (SEM) and probability (*p*-values) were presented to evaluate the effect of cultivar. Correlations between studied parameters were analyzed with Pearson’s correlation test and principal component analysis (PCA) was performed on some of the measured variables (oil content, fatty acids, and bioactive compounds in kernels) using the same statistics program. For the hierarchical clustering analysis, the Euclidean distance estimation was used.

## Results and Discussion

### Proximate Chemical and Mineral Compositions

The pecan kernel proportion and proximate composition in percentages were determined for each cultivar (Table 1). Most of the cultivars reached a kernel weight greater than 50% of the total weight of the nut. Those that are below that threshold correspond to small pecan nuts with the rounded shapes. Apache, Maramec, Oconee, Pawnee, and Sumner cultivars reached the highest percentage of kernel (55.7–58.3%) and Success had the lowest percentage (47.2%).

**TABLE 1:** Percentage of kernel in nuts and proximal composition of pecan kernels from different cultivars.

Cultivar	% Kernel*^A^*	Humidity	Oil	Protein	Ash	Phenols	CH*^B^*
Apache	55,7	3,53	73,54	7,41	1,73	2,62	11,18
Cape Fear	54,0	4,35	69,48	6,38	1,79	2,51	15,50
Desirable	51.6^1^	6,05	65,64	7,13	1,81	2,79	16,59
Elliot	53,0	3,11	70,33	5,47	1,70	1,96	17,43
Gloria grande	50.1^1^	3,85	65,75	7,88	1,84	2,98	17,70
Kiowa	54,6	3,39	69,17	7,19	1,82	3,40	15,03
Mahan	54.3^2^	4,14	70,53	7,19	1,53	3,39	13,05
Maramec	58,3	3,58	69,44	7,13	1,86	2,59	15,39
Nacono	55,2	4,70	67,33	7,63	1,69	2,14	16,51
Oconee	56,7	3,72	68,55	5,00	1,66	3,40	17,66
Pawnee	56.4^1^	3,35	74,69	7,84	1,63	2,19	10,28
Shoshoni	53,2	4,46	67,82	8,94	1,72	2,20	14,76
Starking	54,4	4,05	68,94	6,56	1,55	4,13	14,77
Stuart	48,8	3,38	68,91	5,22	1,73	1,76	18,99
Success	47,2	3,88	69,34	6,44	1,69	2,78	15,87
Sumner	56,6	4,35	68,95	5,78	1,65	3,67	15,61
Average	53,7	3,99	69,28	6,82	1,71	2,78	15,40
SEM*^C^*	1,1	0,25	0,43	0,37	0,05	0,22	0,61
*p*-value*^D^*	< 0.0001	< 0.0001	< 0.0001	< 0.0001	0,0011	< 0.0001	< 0.0001

*^A^% Kernel_As mean of values determined in 3 years, ^1^in 2 years or ^2^in the year of this study. ^B^CH_Carbohydrate content estimated as difference between 100% and the sum of other component analyzed. ^C^SEM, Standard error of mean. ^D^p-values for the effect of the cultivar (ANOVA test).*

Low values of moisture in kernels (3–6%) were observed, with some differences between cultivars. Lower humidity within the nut is important for the best conservation of its seed, so control of humidity during storage is vital for the prevention of fungal growth and an adequate study of its variations and optimal values could be necessary considering cultivar variations found.

Oil contents were between 65 and 75% approximately, although most of the cultivars have values near to 69%. Apache (73.54%) and Pawnee (74.69%) cultivars presented the highest percentages. These results are consistent with those presented by Wakeling et al. (22) for the Australian and United States cultivars, reporting the highest value of 74% of oil in the seed for the Wichita variety. Differences in oil content of pecans have been attributed to diverse factors such as year, location, horticultural practices, and soil type (6, 21). In our research, the significant differences certainly are due to the genetic differences between cultivars. Lipids are not only a source of energy, but in the case of nuts, due to their particular fatty acid profile and lipophilic compounds they content, it has been demonstrated that lipidic bioactive compounds have cardioprotective effects (34).

The pecan cultivars had around 5–9% of the protein content. Value for the pecans grown in the United States and Southern Brazil has been reported to be 7.75/100 g (18) and 10.1% (35), respectively. Its content is directly related to the nitrogen fertilization of the cultivars and this is inversely related to the ratio of unsaturated fatty acids (2). Nitrogen fertilization was not applied in the experimental fields of the pecan cultivars analyzed in this study since mineralization rate of soils was considered sufficient. Levels of nitrogen in leaves (data not shown) were within the normal values, and so it is not surprising that our results for protein content were within the expected range.

The total ash content of kernels varied from 1.53 to 1.86%. These values of ash are in accordance with the mean reported by the USDA (14) but lower than reported in some literature (36). But, relatively high percentages of phenols (1.76–4.13%) and carbohydrates (10.28–19.24%) were found, compared with other studies (8).

Table 2 shows the mineral composition of the pecan kernels in which the predominant elements are potassium, phosphorus, magnesium, and calcium.

**TABLE 2:** Mineral composition of pecan kernels from different cultivars.

Cultivar	K*^A^*	P	Mg	Ca	Mn	Zn	Fe	Cu
Apache	389,18	447,08	157,60	80,42	6,51	5,84	1,83	0,80
Cape Fear	436,88	424,27	181,69	70,12	6,68	4,66	1,37	0,97
Desirable	469,76	460,37	169,11	75,16	6,20	5,36	1,82	0,69
Elliot	419,87	355,27	177,64	61,38	7,95	4,24	1,43	0,80
Gloria grande	464,70	387,80	185,89	57,69	4,38	4,31	1,52	1,33
Kiowa	457,27	351,00	170,67	70,85	4,66	4,90	1,91	1,13
Mahan	402,61	306,75	159,76	57,52	3,54	3,38	1,42	0,91
Maramec	427,77	379,47	170,41	90,02	6,63	5,37	1,16	0,86
Nacono	401,33	388,75	184,75	89,17	4,30	4,18	1,34	0,84
Oconee	410,71	320,93	141,15	67,39	5,47	4,33	1,07	1,01
Pawnee	396,27	344,80	161,08	64,43	4,15	4,90	1,81	1,06
Shoshoni	414,01	312,10	159,23	98,73	3,72	5,12	1,73	1,11
Starking	377,43	332,67	166,36	70,35	4,01	4,06	1,49	0,85
Stuart	447,78	331,75	135,22	61,22	3,31	4,61	1,53	0,94
Success	422,83	320,47	163,50	80,19	3,87	4,11	1,79	1,13
Sumner	424,07	382,66	172,19	63,77	5,47	4,55	1,06	1,02
Average	422,65	365,38	166,02	72,40	5,05	4,62	1,52	0,97
SEM*^B^*	27,91	32,79	12,34	6,14	0,58	0,46	0,16	0,08
*p*-value*^C^*	0,532	0,038	0,258	< 0.0001	< 0.0001	0,086	0,002	< 0.0001

*^A^Mineral content expressed as mg⋅100 g^–1^. ^B^SEM, Standard error of mean. ^C^p-values indicate statistical probability of cultivar effect; (p ≤ 0.05; n = 3) after ANOVA test.*

The average amounts in milligrams for 100 g DW of sample were: K (422.65), P (365.38), Mg (166.02), Ca (72.40), Mn (5.05), Zn (4.62), Fe (1.52), and Cu (0.97). Previous investigations show similar results (22) and confirm that pecan nuts are a good source of minerals given the recommended daily intake values of them.

### Bioactive Compounds and Antioxidant Capacity Analysis in Kernels

The fatty acids profiles reported in Table 3 are consistent with those reported in literature (10). The most abundant fatty acid found was oleic acid between 57 and 64%, followed by the linoleic acid with values between 25 and 31% of the oil. Unsaturated fatty acids have long been recognized by their high-nutritional value and health relevant effects. For example, some studies reported that the fatty acid compositions of tree nuts as pecan, are associated positively with protective effects against oxidative stress-induced neurotoxicity (37), oleic acid has shown cardioprotective effects (38), while linoleic acid has proved to exert hypolipidemic and hepatoprotective roles (39). So, these major fatty acids could contribute to human diet for their important nutraceutical properties. With the values obtained from all the cultivars, a strong inverse relationship was found between oleic and linoleic acids (*r*^2^ = 0.98). These results are in accordance with the reaction of interconversion of those acids discussed by Villareal et al. (6).

**TABLE 3:** Kernel oil fatty acid composition of different pecan cultivars.

Cultivar	C14:0*^A^*	C16:0	C18:0	C18:1	C18:2	C18:3	C20:0	C20:1	SFA*^B^*	MUFA*^C^*	PUFA*^D^*	MUFA/PUFA
Apache	0,04	5,75	2,65	64,47	25,87	0,87	0,12	0,24	8,6	64,7	26,7	2,4
Cape Fear	0,05	6,27	2,31	64,27	25,79	0,98	0,10	0,22	8,7	64,5	26,8	2,4
Desirable	0,05	6,37	2,23	63,13	26,99	0,94	0,11	0,19	8,8	63,3	27,9	2,3
Elliot	0,04	6,03	2,58	63,80	26,32	0,89	0,11	0,23	8,8	64,0	27,2	2,4
Gloria grande	0,06	7,57	2,12	58,27	30,44	1,31	0,08	0,14	9,8	58,4	31,7	1,8
Kiowa	0,05	6,92	2,41	58,80	30,22	1,29	0,11	0,22	9,5	59,0	31,5	1,9
Mahan	0,06	7,68	2,74	60,89	27,45	0,94	0,09	0,14	10,6	61,0	28,4	2,1
Maramec	0,05	6,94	2,37	63,02	26,51	0,79	0,11	0,21	9,5	63,2	27,3	2,3
Nacono	0,05	6,55	2,28	61,26	28,66	0,94	0,10	0,17	9,0	61,4	29,6	2,1
Oconee	0,06	7,36	2,34	58,69	30,13	1,07	0,12	0,23	9,9	58,9	31,2	1,9
Pawnee	0,05	7,13	2,27	64,28	24,85	1,11	0,10	0,21	9,6	64,5	26,0	2,5
Shoshoni	0,06	7,03	2,19	63,32	26,11	1,09	0,07	0,14	9,4	63,5	27,2	2,3
Starking	0,07	8,52	2,06	59,16	28,97	1,00	0,08	0,14	10,7	59,3	30,0	2,0
Stuart	0,06	7,51	2,00	59,33	29,67	1,13	0,09	0,21	9,7	59,5	30,8	1,9
Success	0,05	6,01	2,16	62,92	27,64	0,90	0,10	0,23	8,3	63,1	28,5	2,2
Sumner	0,07	8,24	2,29	57,03	31,06	1,08	0,09	0,16	10,7	57,2	32,1	1,8
Average	0,05	6,99	2,31	61,41	27,92	1,02	0,10	0,19	9,5	61,6	28,9	2,1
SEM*^E^*	0,01	0,20	0,04	0,47	0,43	0,03	0,01	0,02				
*p*-value*^F^*	0,007	< 0.0001	< 0.0001	< 0.0001	< 0.0001	< 0.0001	0,018	< 0.0001				

*^A^Fatty acid expressed as percentage of total fatty acid content. Myristic acid (C14: 0), Palmitic acid (C16: 0), Stearic acid (C18: 0), Oleic acid (C18: 1), Linoleic acid (C18: 2), Linolenic acid (C18: 3), Arachidic acid (C20: 0), Eicosenoic acid (C20: 1); ^B^SFA: Saturated fatty acids; ^C^MUFA: Monounsaturated fatty acids; ^D^PUFA: Polyunsaturated fatty acids. ^E^SEM: standard error of mean; ^F^p-values indicate statistical probability of cultivar effect (p ≤ 0.05; n = 3) after ANOVA test.*

The kernels of the pecan cultivars studied are low in the saturated fatty acids and high in unsaturated. Regarding MUFA and PUFA values, and also the ratio between both (MUFA/PUFA) in our research are consistent with other reports (40). Cultivars with the lower ratio are more prone for oxidation, so this can be considered as an indicator of the quality of kernels and it could be used as a selection factor for high resistance to rancidity in storage conditions of nuts after harvest. Some authors also reported differences in fatty acid composition between pecan varieties, as well as due to year of production, cultivation sites, crop management and cultivars studied, and factors such as soil and climate (6, 22, 41, 42).

The total extractable phenolic content, condensed tannin content, tocopherols, and antioxidant activity (ORAC) were significantly affected by the pecan cultivar (Table 4).

**TABLE 4:** Total extractable phenolic compounds, condensed tannins, tocopherols, and antioxidant activity in kernels of different cultivars.

Cultivar	Phenols*^A^*	Tannins*^B^*	γ – Tocopherol*^C^*	β – Tocopherol*^C^*	ORAC*^D^*
Apache	26,18	4,87	106,40	17,20	97,05
Cape fear	25,14	6,23	117,10	22,13	115,00
Desirable	26,23	2,41	126,93	13,90	156,60
Elliot	19,61	5,42	139,50	23,63	33,60
Gloria grande	29,75	4,15	95,97	15,57	39,00
Kiowa	34,02	7,24	118,07	22,50	92,85
Mahan	33,91	7,53	119,77	21,47	50,80
Maramec	25,92	7,34	101,53	15,73	131,95
Nacono	21,36	10,29	106,47	16,97	61,50
Oconee	34,04	10,70	102,20	14,60	58,95
Pawnee	21,95	7,10	101,47	20,17	145,15
Shoshoni	22,01	5,78	133,83	18,37	64,20
Starking	41,30	11,69	130,17	15,27	67,65
Stuart	17,60	4,01	117,33	23,60	23,15
Success	27,76	5,57	111,57	21,70	44,45
Sumner	36,65	10,78	117,00	15,20	23,47
Average	27,71	6,94	115,33	18,63	75,34
SEM*^E^*	2,13	0,89	5,48	1,29	11,29
*p*-value*^F^*	< 0.0001	< 0.0001	< 0.0001	< 0.0001	< 0.0001

*^A^Phenols: Total extractable phenolics compounds as mg GAE⋅g^–1^ kernel; ^B^Tannins: Condensed tannins as mg CE⋅g^–1^ kernel; ^C^g and b-tocopherol as mg of α-tocopherol⋅g^–1^ kernel; ^D^ORAC: Antioxidant capacity as μmol Trolox⋅g^–1^ kernel. Different letters in the same column indicate significant differences according to Tukey test (p ≤ 0.05). ^E^SEM: standard error of mean; ^F^p-values indicate statistical probability of cultivar effect (p ≤ 0.05; n = 3) after the ANOVA test.*

Phenolic compounds ranged from 17.60 to 41.30 mg GAE/g kernel in Stuart and Starking cultivars, respectively. These results were above the reported values of total phenols in pecan (10). The range varies from significantly lower values than pecans from Mexico (12.1 mg GAE/g average) (20) or USDA database (20.1 mg GAE/g) (14) to much higher than those in pecans cultivated in China (up to 29.5 mg GAE/g) (43). Regarding solvent, extraction and analysis conditions, recovery of total phenolic compounds may be affected, for example, ultrasonic assisted extraction used in our study seems to improve extraction yields than those obtained with other methods. It has been reported that nut phenolics locate predominantly in the kernel skin, therefore, differences in total phenolics between cultivars may be also due to differences in the content of skin (15). Consequently, more investigations about kernel skin proportion and composition could be necessary.

Condensed tannin values (2.41–11.69 mg catechin/g kernel) were lower than those reported in literature (20–30 mg catechin/g kernel) (10). Desirable cultivar presented the lower CT value while Nacono, Oconee, Starking, and Sumner the higher ones with significant differences between the cultivars.

The content of γ -tocopherol among the cultivars ranged from 95.97 to 139.50 mg γ -tocopherol/g of kernel and was predominant respect to β -tocopherol contents found (13.30–23.63 mg/g). These results are important since tocopherols are the main components of vitamin E complex. Some studies report that γ -tocopherol is more potent as antioxidant, anti-inflammatory, and cardioprotective than its homolog α-tocopherol, having special interest its effect on chronic diseases (16).

The consumption of pecan nuts and their effects on the oxidative stress have been reviewed based on both *in vitro* and *in vivo* studies. In fact, large variation in antioxidant activity *in vitro* analysis was found between kernels of different cultivars, with cultivars such as Desirable, Pawnee, and Maramec of the higher antioxidant capacity in accordance to the range reported in the literature (132–157 μmol Trolox/g pecan) (19, 20), and on the other hand, the cultivars such as Stuart and Sumner with the lowest. Table 5 shows a positive correlation between phenolic compounds and ORAC values (*r*^2^ = 0.62; *p* < 0.01), along with γ-tocopherol phenolic compounds (*r*^2^ = 0.80; *p* < 0.01), and tannins (*r*^2^ = 0.73; *p* < 0.01). So, the contribution of vitamins, tannin, and other bioactive compounds should also be taken into consideration in antioxidant capacity potential of the pecan nuts (13).

**TABLE 5:** Matrix of Pearson’s Correlations between the variables evaluated from kernels **(A)** and shells **(B)** for the sixteen pecan cultivars.

(A)	γ – Tocopherol	β – Tocopherol	Phenols	Tannins	ORAC
γ – Tocopherol	1	0,02	**0,80**	**0,73**	0,35
β – Tocopherol		1	0,06	0,05	0,16
Phenols			1	0,00	**0,62**
Tannins				1	0,44
ORAC					1

**(B)**	**Phenols**	**Tannins**	**Anthocyanins**	**ORAC**	

Phenols	1				
Tannins	**0,78**	1			
Anthocyanins	0,53	0,53	1		
ORAC	**0,59**	0,41	0,33	1	

*Values in bold indicate statistical correlations at p ≤ 0.05.*

**FIGURE 2:**
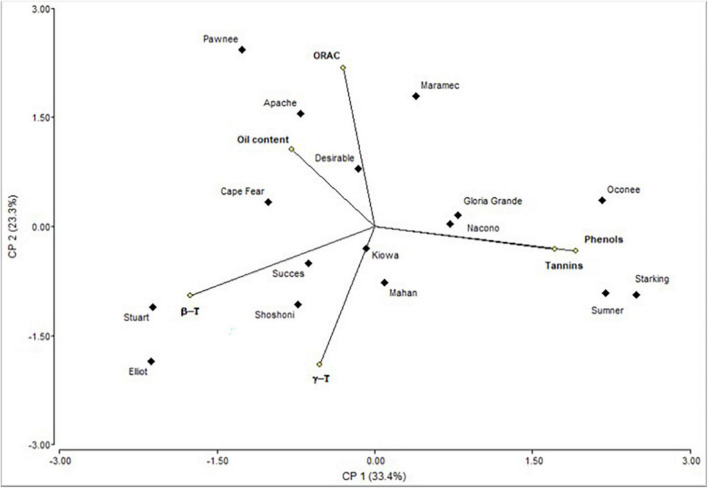
Biplot representation of the scores of the principal component analysis of the bioactive compounds determined in kernel of cultivars.

**FIGURE 3:**
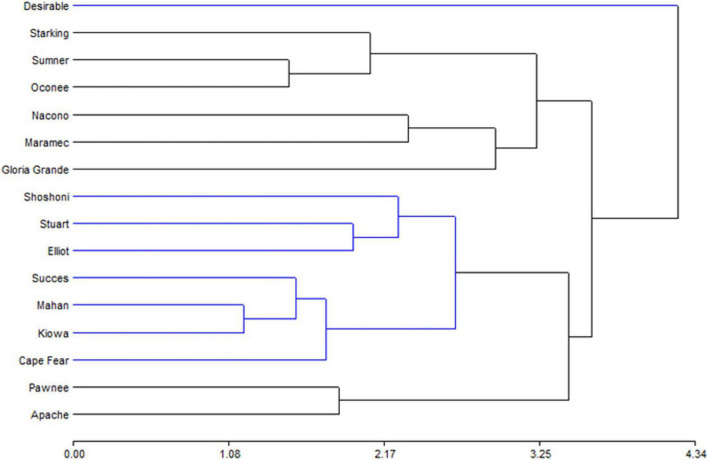
Hierarchical cluster analysis representation of the bioactive compounds determined in kernel of cultivars.

The biplot produced by PCA is presented to facilitate the interpretation of the results (Figure 2). Oconee, Starking, and Sumner cultivars were grouped, characterized by high levels of phenols and tannins. On the other hand, Elliot and Stuart were characterized by high concentrations of tocopherols. However, other cultivars (Apache, Maramec, and Pawnee) were distinguished by high-antioxidant activity and oil content. Complementary to the PCA, the hierarchical cluster analysis (HCA) dendrogram showed that samples were separated (Euclidian distances) in at least two main groups, and several subgroups (Figure 3). One of these subgroups were produced by Starking, Summer, and Oconee, associated with phenols and tannin. On the other extreme, Pawnee and Apache group was explained by the oil content. This confirms the high variability in the bioactive content among pecan cultivars because of the genetic factor since all were grown under the same climatic conditions and cultural management. Similar results were observed in the pecan nuts cultivars in Southern Brazil (44), although a different phytochemical characterization was evaluated.

### Pecan Shells Composition and Antioxidant Capacity

The average of extractable phenolic compounds from nut shells of the cultivars evaluated were 2–3 times higher than those found in kernels (Table 6).

**TABLE 6:** Total extractable phenolic compounds, condensed tannins, anthocyanins, and antioxidant activity in shells of different cultivars.

Cultivar	Phenols*^A^*	Tannins*^B^*	Ant^C^	ORAC*^D^*
Apache	116,41	326,27	1,54	578,88
Cape fear	52,24	185,58	1,57	321,98
Desirable	66,12	220,87	1,39	262,03
Elliot	32,18	130,03	1,09	57,15
Gloria grande	97,69	270,90	2,92	547,43
Kiowa	33,04	130,32	1,23	125,13
Mahan	70,10	221,24	1,29	207,48
Maramec	78,29	221,01	2,03	359,07
Nacono	109,96	328,64	1,63	474,16
Oconee	90,04	189,64	1,81	516,34
Pawnee	116,74	357,49	3,16	421,75
Shoshoni	59,42	207,87	2,16	166,92
Starking	51,12	240,32	1,54	309,10
Stuart	99,38	275,14	3,03	529,85
Success	53,66	135,92	1,06	508,87
Sumner	49,77	222,74	1,49	224,21
Average	73,51	229,00	1,81	350,65
SEM*^E^*	8,35	29,58	0,67	54,78
*p*-value*^F^*	< 0.0001	0,0008	0,2215	< 0.0001

*^A^Phenols: Total extractable phenolics compounds as mg GAE⋅g^–1^ shell; ^B^Tannins: Condensed tannins as mg CE⋅g^–1^ shell; ^C^anthocyanins as mg of c3g⋅g^–1^ shell; ^D^ORAC: Antioxidant capacity as μmolTrolox⋅g^–1^ kernel. Different letters in the same column indicate significant differences according to Tukey test (p ≤ 0.05). ^E^SEM: standard error of mean; ^F^p-values indicate statistical probability of cultivar effect; (p ≤ 0.05; n = 3) after ANOVA test.*

Average of condensed tannins of the shells (229.00 mg CE/g pecan shell) was also higher than that in the kernels but in a factor of 30. Considering that storage and processing conditions affect leaching of tannins from shells to kernels, the content of CT in shell is an important factor in the cultivars selections, mainly due to the protection function of high concentration of tannins (6). The range values obtained was 130.03–357.49 mg c3 g/g shell. Elliot, Kiowa, and Success shells showed the lowest values while Apache, Nacono, and Pawnee the highest values. Total anthocyanins content ranged between 1.35 and 3.90 mg c3 g/g of shell with Success and Pawnee cultivars with the lower and higher values, respectively. Total anthocyanins contents are considered high if it is referred to as a secondary by-product of the pecan industry, and then pecan shells could be a good source of natural dyes. Significant high correlations were found (Table 5) between shells phenols with tannins (*r*^2^ = 0.78; *p* < 0.0001) and with anthocyanins contents (*r*^2^ = 0.53; *p* = 0.01). Antioxidant activity measured by hydrophilic ORAC were higher in the Apache cultivar and lower in the Elliot. ORAC also showed higher values in shells than in kernel for all cultivars in a factor of five, in accordance with reports in literature (20). Significant correlations were found between antioxidant activity and total phenolic compounds (*r*^2^ = 0.59, *p* < 0.01); tannins (*r*^2^ = 0.56, *p* = 0.02), but not for anthocyanins (*r*^2^ = 0.33, *p* = 0.07).

**FIGURE 4:**
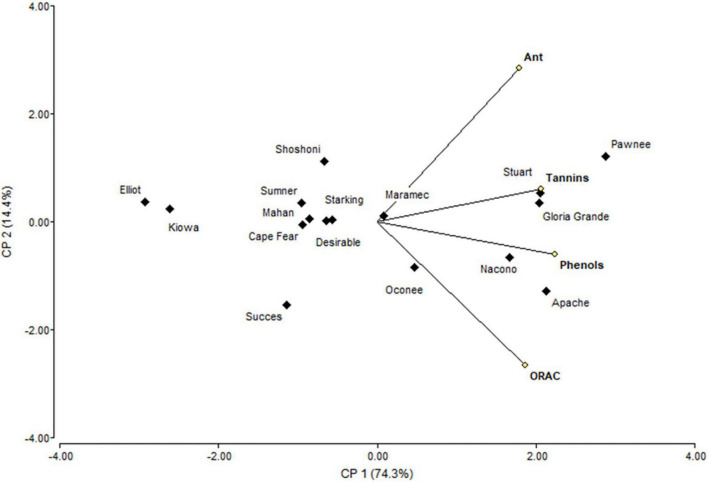
Biplot representation of the scores of the principal component analysis of the bioactive compounds determined in shell of cultivars.

**FIGURE 5:**
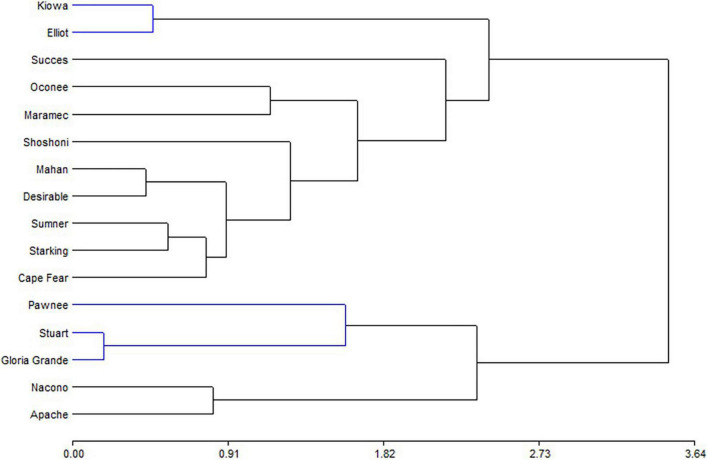
Hierarchical cluster analysis representation of the bioactive compounds determined in shell of cultivars.

Principal component analysis (Figure 4) showed that PC1 explain 74.3% of variability, separating tannin and phenols from anthocyanins and ORAC. Shells from Pawnee, Stuart, and Gloria Grande were associated with tannins, while Nacono and Apache are related to phenols content. Another big group of nine cultivars (Elliot, Kiowa, Success, Cape Fear, Desirable, Mahan, Summer, Starking, and Shoshoni) was generated but remained dissociated with the variables measured in shells. The similarity dendrogram showed that samples were separated (Euclidian distances) in two main groups, and several subgroups (Figure 5). Kiowa and Elliot were one of the closest subgroups, likewise Nacono and Apache cultivars. The grouping of Kiowa and Elliot could be explained by their phenols and ORAC values as shown in Figure 4. In contrast, Nacono and Apache are not explained by the variables examined.

The combination of different antioxidant *in vitro* assays are recommendable (e.g., DPPH and ORAC) to supply a reliable assessment of the antioxidant properties of fruits and vegetables, but it is not possible to asseverate absolute values (45). Despite that, the values of relative antioxidant activity between cultivars and the contribution of specific antioxidant compounds could be studied in the future research. Furthermore, different analytical approaches are required to characterize the antioxidant capacity and the correlation with the lipophilic bioactive compounds, such as tocopherols. Furthermore, it is remarkable that some genotypes evaluated in these particular conditions of Uruguay have a high level on nutraceuticals and potential antioxidant capacity. Other studies have shown that the importance of genetic variability, locations, or cultivation conditions on chemical composition of pecan nut (8, 41, 44), indicating that each country needs to establish its own data for nutritional purposes.

Specific edaphoclimatic conditions are one of the components that determines the performance and results of each cultivar. The specific soil depth and slow water infiltration combined with a very irregular rain pattern submits plants to different stresses (excess and deficit), (5) which could be of key importance to promote secondary metabolism and the obtention bioactive compound in nuts. In a recent study at the same location, our group set experiments to study the impact of clime, soil, and irrigation management in olive fruits chemical composition (46). Similar research approach could be useful to understand mechanisms and determine thresholds for pecan bioactive compounds accumulation.

## Conclusion

To our knowledge, this is the first report in the literature showing the bioactive composition and antioxidant capacity potential of the sixteen cultivars grown in an experimental orchard under identical agricultural conditions. Although bioactive composition and antioxidant activity may vary widely depending upon the extraction techniques and solvents used, some features in the chemical profiles may be demonstrated for pecan cultivars grown in Uruguay. Proximate chemical composition, minerals, phenolics, and tocopherols were similar to other world regions where pecan nuts are cultivated. The balanced content of bioactive compounds described, suggests that pecan kernels could be a valuable diet source of minerals and natural antioxidants with demonstrated health beneficial effect. This should be systematically investigated and will be useful for different purposes in the industry, to inform consumers about the greater potential health benefits of consuming nuts and to cover nutritional or nutraceutical diet requirements. The high heterogeneity found is desired in commercial crop species, represents an important source of genetic variation, and can be exploited toward further breeding selection of the pecan crops. Despite its limitations because the field plot design has only duplicates, this study established a complete biochemical profile showing the importance of genetic variability and reinforcing the need of deeper studies of the effect of cultural, agronomic, and climatic factors in the nutritional composition for different cultivars. In addition, pecan shell has been shown to have a great potential as a raw material to develop by-products also with high nutritional and nutraceutical value either for food or feed.

## Data Availability Statement

The raw data supporting the conclusions of this article will be made available by the authors, without undue reservation.

## Author Contributions

FI and RZ: conceptualization. GG and VF: chemical analysis, statistical analysis, and writing—original draft preparation. VF, GG, HH, RZ, and FI: writing—review and editing. FI, HH, and RZ: funding acquisition. All authors have read and agreed to the published version of the manuscript.

## Conflict of Interest

The authors declare that the research was conducted in the absence of any commercial or financial relationships that could be construed as a potential conflict of interest.

## Publisher’s Note

All claims expressed in this article are solely those of the authors and do not necessarily represent those of their affiliated organizations, or those of the publisher, the editors and the reviewers. Any product that may be evaluated in this article, or claim that may be made by its manufacturer, is not guaranteed or endorsed by the publisher.
